# Postoperative Hip Center Position Associated With the Range of Internal Rotation and Extension During Gait in Hip Dysplasia Patients After Total Hip Arthroplasty

**DOI:** 10.3389/fbioe.2022.831647

**Published:** 2022-02-28

**Authors:** Yi Hu, Diyang Zou, Qi Sun, Mengda Jiang, Huiwu Li, Tsung-Yuan Tsai, Jingwei Zhang

**Affiliations:** ^1^ Department of Orthopaedic Surgery, Shanghai Ninth People’s Hospital, Shanghai Jiao Tong University School of Medicine, Shanghai, China; ^2^ School of Biomedical Engineering, Med-X Research Institute, Shanghai Jiao Tong University, Shanghai, China; ^3^ Department of Radiology, Shanghai Ninth People’s Hospital, Shanghai Jiao Tong University School of Medicine, Shanghai, China; ^4^ TaoImage Medical Technologies Corporation, Shanghai, China

**Keywords:** total hip arthroplasty, developmental hip dysplasia, kinematics, fluoroscope, hip center, gait range of motion

## Abstract

**Background:** Total hip arthroplasty (THA) for hip dysplasia patients is sometimes complex and compromises pathomorphological changes in these patients. However, it remains unclear whether it is preoperative deformities or postoperative structures or anatomic changes during THA that have the most remarkable correlation with the hip dynamic function during gait. The purpose of this study was to investigate this relationship and propose insights into the surgical reconstruction strategy in patients with developmental dysplasia of the hip.

**Methods:** A total of 21 unilateral hip dysplasia patients received computed tomography scans for the creation of 3D hip models before surgery and at the last follow-up. Acetabular and femoral orientations, hip center positions, and femoral length were measured before and after THA. Hip kinematics of the operated side during gait was quantified using a dual fluoroscopic imaging technique. Pearson correlation and multiple linear regression were performed to evaluate the relationship between hip maximum range of motion in six directions and demographics characters and above hip anatomic parameters before and after THA and their changes in surgery.

**Results:** Pearson correlation analysis found significant correlations with the gait range of motion mainly in postoperative structures, including postoperative hip center positions and acetabulum and combined anteversion. Further multiple linear regression indicated that a laterally placed hip center was significantly correlated with an increased internal rotation (*R* ^2^ = 0.25, *p* = 0.021), which together with increased postoperative acetabulum anteversion explained 45% of external rotation decreasing (*p* = 0.004). A proximally placed hip center was correlated with more extension (*R* ^2^ = 0.30, *p* = 0.010). No significant demographic characters or preoperative deformities or surgical changes were included into other multiple regression models.

**Conclusion:** Strong correlations between postoperative structures, especially hip center positions and gait range of motion in unilateral hip dysplasia patients after THA were found. It indicated that postoperative prosthesis structures, particularly hip center positions had significant impact on the hip gait motion range and should be treated with particular caution in surgery.

## Introduction

Symptomatic osteoarthritis secondary to developmental dysplasia of the hip (DDH) is a common indication for total hip arthroplasty (THA) (Cooperman et al., 1983; Harris, 1986; Hartofilakidis et al., 1996). Surgical reconstructions of appropriate component alignments, anatomic acetabular rotation center, and equal leg length are essential requirements in THA that guarantee good clinical results (Galia et al., 2017). However, a broad range of pathomorphological changes from both the acetabulum and femoral sides exist in DDH patients, including bony acetabular defect, a high-riding or even dislocated femoral head, and excessively anteverted femur. Such deformities sometimes make THA in DDH patients a highly complex reconstruction with a higher risk of complications, and some surgical reconstruction goals have to be compromised (Crowe et al., 1979; Biant et al., 2009; Galea et al., 2018; Hitz et al., 2018).

How to best restore the hip anatomy and improve the hip function under various pathomorphology changes in DDH patients remains challenging. Although anatomic hip center reconstruction is required for biomechanical superiority in THA, cup coverage is insufficient due to acetabular defects in DDH patients, and surgeons sometimes have to put the cup at a slightly superior place (Xu et al., 2016). Restoration of equal leg length contributes to symmetry gaits after THA, while in DDH patients with subluxated or high-riding hip centers, deeper stem fixation or femoral shortening may be necessary to reduce the hip to avoid potential neurovascular injury (Li et al., 2018). The Lewinnek safe zone serves as a valuable tool for guiding prosthesis alignments for conventional THA (Lewinnek et al., 1978). However, in DDH patients with variant acetabulum and femur, appropriate prosthesis alignments vary with individuals, and it is quite a challenge to adjust in operation ((Robertson et al., 1996). When standard reconstructions become difficult in THA for DDH patients, chasing for anatomic reconstruction regardless of pathomorphological changes or diminishing surgical changes and compensating for preoperative morphology deserves further investigation. When not all pathomorphological changes can be reconstructed, which structures should be anatomically built with priority also need to be clarified.

Different reconstruction strategies in THA for DDH patients inevitably lead to different biomechanical environments from standard THA (Johnston et al., 1979; Hartofilakidis and Karachalios, 2004). Postoperative gait kinematics is a reflection of such biomechanical characters and is closely related to clinical outcomes (Karaismailoglu et al., 2019a). Understanding how surgical reconstruction strategies influence postoperative gait kinematics is crucial to make necessary adjustments for better kinematics patterns. Previous studies primarily used skin-marker-based gait analysis to study such correlations. Karaismailoglu investigated the impact of hip center height by gait analysis and found that a unilateral 15-mm superiorly placed hip center measured on an X-ray-reduced hip motion range and increased the fall risks, while such effect disappeared in bilateral high hip center patients (Karaismailoglu et al., 2019a; Karaismailoglu et al., 2019b). Harris gait scores were reduced if the cups were implanted over 75 mm higher than the interteardrop line (Berninger et al., 2019). Although leg length discrepancy was within 20 mm on radiographs, unilateral DDH patients showed less gait symmetry than healthy controls (Chen et al., 2018). However, there was a lack of literature precisely measuring hip structures and gait motions and testing their comprehensive relationship.

In this study, we first used a dual fluoroscopic imaging system (DFIS) based on 3D computed tomography (CT) modeling to measure *in vivo* six-degrees-of-freedom (6-DOF) hip kinematics of DDH patients after THA during gait. Then, we tested the relationship between hip maximum range of motion (ROM) during gait and demographics characters and hip anatomic parameters before and after THA and their changes in surgery. We aimed to answer: 1) the relationship between aforementioned parameters and gait range of motion in such DDH patients; 2) whether demographics characters or preoperative or postoperative anatomic structures or their changes in surgery had the greatest influence on gait range of motion and which one should be treated with precaution during THA.

## Materials and Methods

### Patients Demographics

Our Institutional Review Board approved this study. Written consent was obtained from each participant before taking part in this study. Patients were bilaterally affected or with diseases affecting joints movements or history of THA dislocation or periprosthetic fractures were excluded. A total of 21 patients (five males and sixteen females) with a good functional unilateral THA (Harris hip score >90 points) for end-stage osteoarthritis secondary to DDH and, the other side, healthy were enrolled. A total of nine of them were diagnosed as Crowe II, six as Corwe III, and the rest six as Crowe IV(5). Their average age was 58.4 (±13.7, range 25.3–74.3) years; average height and body mass index (BMI) were 1.62 m (±0.06, range 1.53–1.73) and 23.4 (±2.0, range 19.3–27.6), respectively. The average follow-up period was 4.4 years (±2.3, range 2.0–11.7). THA was performed by the same group of experienced surgeons using the posterolateral approach. Normally, the acetabular cup was inserted with press-fit fixation in anatomic or slightly superior place, and necessary deeper stem fixation was performed when it was difficult to reduce. Capsule was cut off in highly dislocated patients for hip reduction. Muscles and soft tissue release was reduced to the least. No release was made to these patients’ gluteus maximus or the iliopsoas. One patient underwent femoral shortening osteotomy in this cohort. Cementless cups (SecurFix, Stryker, United States, and Pinnacle, DePuy, United States) and stems (SecurFix, Stryker, United States; Corail, DePuy, United States, and S-ROM, DePuy, United States) were implanted for all patients.

### CT-Based 3D Modeling and Anatomical Parameter Measurements

All patients received CT scans (Siemens, SOMATOM Definition Flash, Germany) covering the bilateral anterior iliac spine and the posterior borders of the medial and lateral condyles with 0.5-mm interspacing thickness before and after THA. The CT images were segmented in Amira (Amira 6.7, Thermo Fisher Scientific, Rockford IL, United States) to create the 3D surface models of the pelvic and bilateral femur before and after THA as well as the models of the implanted prosthesis.

**FIGURE 1:**
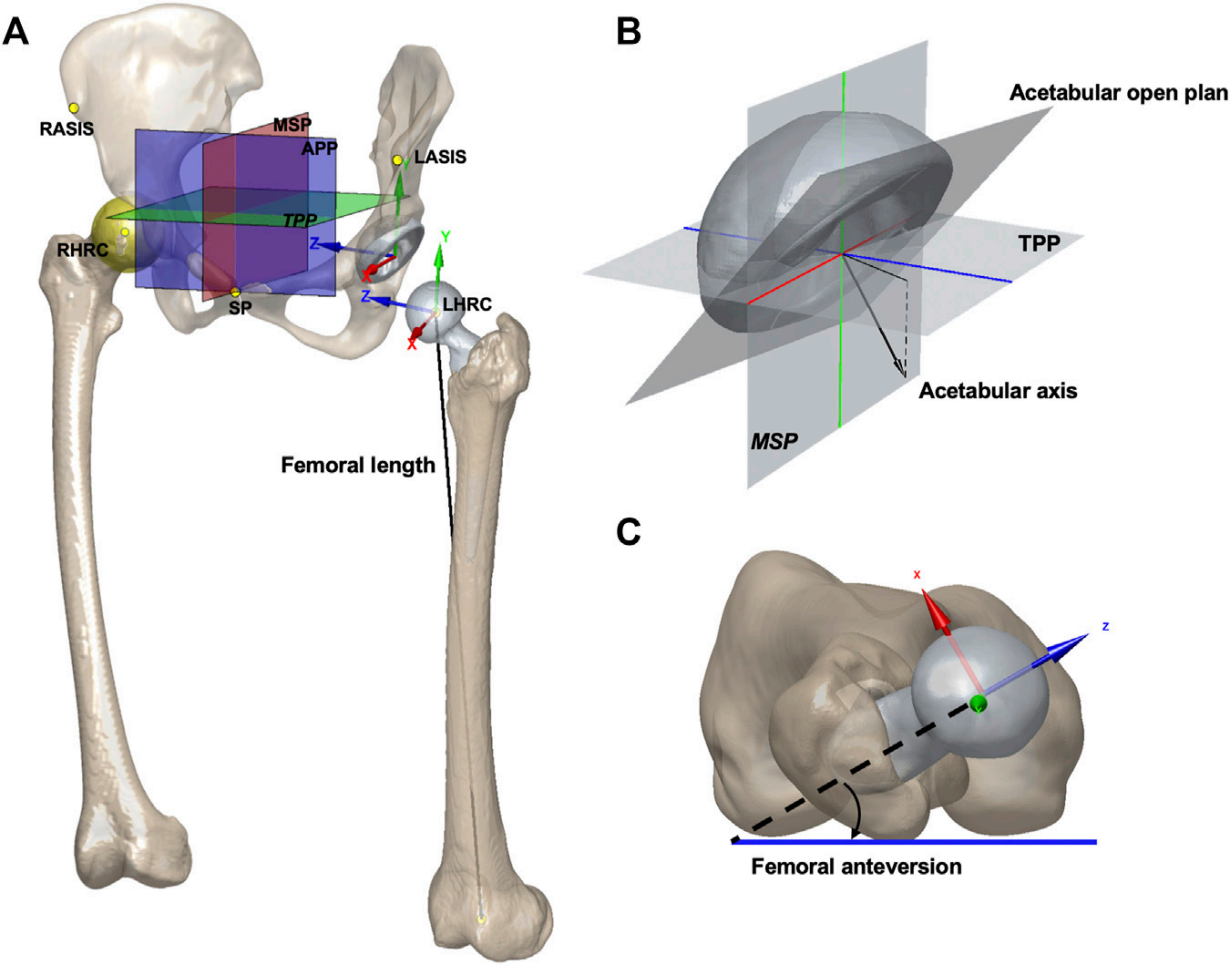
**(A)** APP, TPP, MSP, and hip rotation center were shown on models of the pelvis and implants. The femoral length was calculated as the absolute length. The origin of the pelvic coordinate system was at the center of the acetabular cup. The pelvic *z*-axis was parallel to a line connecting the right and left ASISs and pointing to the right. The pelvic *x*-axis was parallel to a line lying in the plane defined by the two ASISs and the midpoint of the two posterior–superior iliac spines, orthogonal to the *Z*-axis, and pointing anteriorly. The origin of the femoral coordinate system was at the center of the femoral head. The femoral *y*-axis was parallel to the long axis of the proximal femoral shaft. The *x*-axis was parallel to the normal vector of the plane formed by the *y*-axis and the center of the femoral head. The pelvic and femoral local coordinate systems were defined for describing the hip joint rotations. SP: symphysis pubis. **(B)** Acetabular open plane and axis are shown. The cup anteversion was defined as the angle between the transverse axis (blue) and the acetabular axis projected onto the TPP. The cup inclination was defined as the angle between the longitudinal axis (green) and the acetabular axis. **(C)** Measurement for femoral anteversion is shown.

The anterior pelvic plane (APP), established by the anterior–superior iliac spine (ASIS) and pubic symphysis, was used as the reference plane to evaluate anatomical acetabular orientation. The transverse pelvic plane (TPP) was set perpendicular to APP and horizontal to the connecting between the left and right ASIS. The medial sagittal plane (MSP) was set perpendicular to APP and TPP and passed the midpoint of pubic symphysis (Figure 1A). Anatomical cup anteversion and inclination of both sides were obtained on 3D models following Murray’s definition (Murray, 1993). The cup anteversion was measured as the angle between the transverse axis and the acetabular axis projected onto the TPP, with cup inclination as the angle between the longitudinal axis and the acetabular axis (Figure 1B). The hip rotation center (HRC) was determined as the center of the best fit sphere to the femoral head. The relative distance of the HRC to APP, TPP, and MSP compared to that of the healthy side was recorded as HRC position, and positive meant a more proximally, laterally, or anteriorly placed HRC. The femoral anteversion was defined as the angle formed by the femoral neck axis and the plane passing posterior medial and lateral femoral condyles and the lesser trochanter (Figure 1C). Combined anteversion was the sum of acetabular and femoral anteversion (Zhang et al., 2014). Femoral length was calculated as the absolute length of the distance between HRC and the midpoint of femoral condyles (Figure 1A). The values of these variables before and after THA and their changes in THA as well as demographic variables including age, height, and BMI were all tested as possible contributing factors to postoperative kinematics.

### Dual Fluoroscopic Imaging System and Hip Gait Kinematics Measurements

**FIGURE 2:**
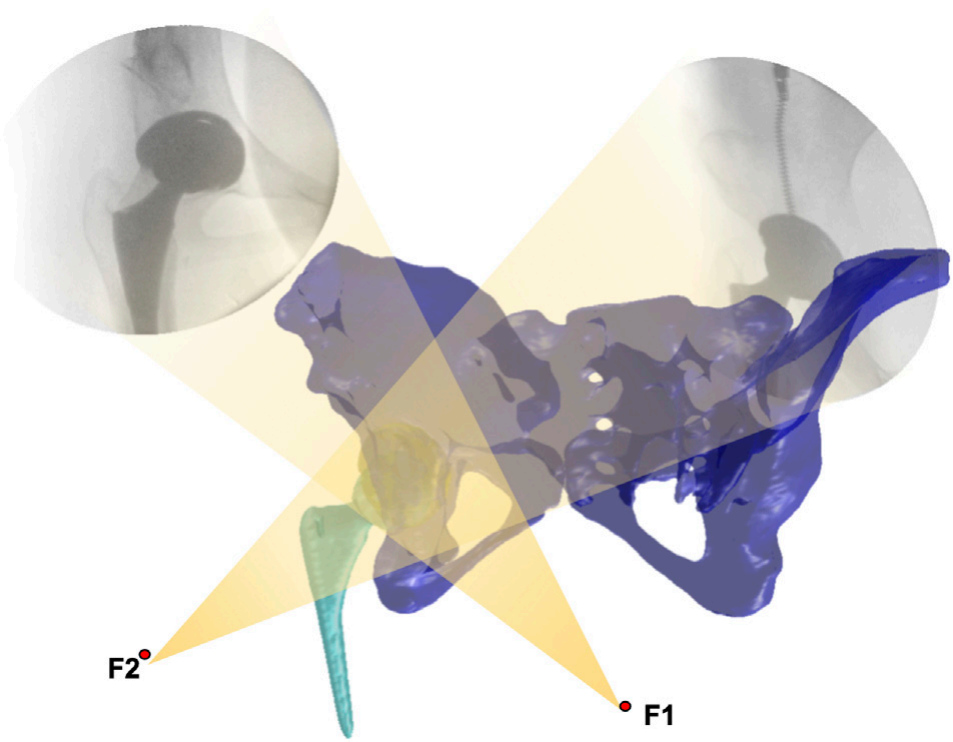
Virtual environment of the dual fluoroscopic imaging system.

Each participant’s operated hip was simultaneously imaged using two fluoroscopes (ARCADIS Varic, Siemens, Germany) under snapshots (with an 8-ms pulse width, 60–80 kV, and 0.042–0.066 mAs) while walking on a treadmill at a speed of 1 km/h. The 3D surface models were then imported into a customized program in MATLAB (2020a, MathWorks, Natick, MA, United States). The pelvic and femoral local coordinate system was defined following the International Society of Biomechanics recommendation (Wu et al., 2002). Next, local coordinate systems together with fluoroscopic images and 3D surface models were imported into a simulated environment in MATLAB (Figure 2), in which the 3D joint models could be translated and rotated through 6-DOF in the 3D virtual space until the models matched the fluoroscopic images. The tracking error for this technique is less than 0.35°mm and 0.55° in calculating hip joint translations and rotations (Tsai et al., 2013). The maximum range of flex-extension, add-abduction, and axial rotation was calculated for further analysis.

### Statistical Analysis

All statistical analyses were performed by SPSS for Mac (version 26.0; SPSS, Chicago, IL, United States). Pearson’s correlation was used to analyze the relationship of various linear variables including demographic variables and preoperative and postoperative component positions as well as their changes in surgery with postoperative 6-DOF ROM of the affected hip. Forward stepwise multiple regression analysis was conducted to determine possible contributing factors. The level of significance was defined as *p* < 0.05.

## Results

### Postoperative Gait ROM and Hip Anatomic Parameters

On an average, the flexion degree was 30.4 ± 9.7°, and the extension degree reached −2.6 ± 6.8°. The detailed 6-DOF ROM was described in Table 1. The average cup and combined anteversion and femoral length were increased, and cup inclination and femoral anteversion were decreased after surgery (Table 2). HRC was reconstructed in a more relatively distal, medial, and posterior place with an average translation of 9 mm, 15.7, and 2.1 mm, respectively.

**TABLE 1:** Maximum 6-DOF range of motion during gait.

Patient ID	Flexion (°)	Extension (°)	Abduction (°)	Adduction (°)	ER (°)	IR (°)
1	37.9	−11.3	3.7	4.5	15.9	−5.2
2	29.5	−2.3	3.7	4.3	5.3	−1.3
3	24.2	0.5	4.6	2.4	2.1	13.5
4	33.6	−0.9	6.7	2.8	26.7	−12.6
5	39.1	−6.8	5.8	4.1	14.6	4.7
6	29.9	4.2	2.3	5.0	19.4	0.9
7	51.0	1.3	4.3	4.8	27.3	0.5
8	27.4	2.6	−0.1	2.2	10.6	1.3
9	37.5	−5.7	2.1	8.2	16.2	−4.8
10	17.1	2.7	5.0	1.9	14.0	3.7
11	30.3	7.2	4.4	3.5	20.3	0.6
12	42.8	−21.2	3.5	4.1	24.1	−23.2
13	15.4	0.3	3.1	3.9	7.0	4.1
14	15.2	8.1	3.3	5.0	−3.5	14.3
15	20.9	−1.6	3.9	4.5	2.1	2.1
16	34.1	−10.8	0.6	4.5	14.3	−2.2
17	22.0	−5.1	2.1	4.9	−0.4	12.3
18	30.0	1.1	1.6	3.9	5.8	−1.0
19	28.9	−3.3	2.4	6.2	10.8	1.7
20	46.0	−7.6	4.5	0.9	25.0	−4.4
21	25.1	−6.4	−0.2	2.3	10.7	−2.2
Average	30.4	−2.6	3.2	4.0	12.8	0.1
SD	9.7	6.8	1.8	1.6	9.0	8.3

a A negative value meant that the maximum motion degrees in certain direction during gait were still smaller than those in a static standing position. ER, external rotation; IR, internal rotation.

**TABLE 2:** Hip anatomic parameters measured before and after THA and their changes in surgery.

	Acetabulum ante. (°)			Acetabulum inc. (°)			Femoral ante. (°)			Combined ante. (°)			HRC P/D^*^ (mm)			HRC A/P^*^ (mm)			HRC M/L^*^ (mm)			Femoral length* (mm)			
	Pre	Post	D	Pre	Post	D	Pre	Post	D	Pre	Post	D	Pre	Post	D	Pre	Post	D	Pre	Post	D	Pre	Post	D	
**Max**	38.6	50.2	27.2	79.1	51.0	−7.1	51.2	41.9	20.0	89.9	64.6	31.2	53.8	19.7	6.8	22.7	20.6	3.1	35.9	6.7	6.2	12.5	10.4	24.5	
**Min**	1.0	9.3	−19.0	49.9	39.4	−39.7	8.4	7.5	−25.5	25.0	27.6	−34.4	−13.9	−23.9	−38.8	−25.1	−29.3	−8.9	−9.9	−17.0	−37.8	−30.9	9.9	−15.9	
**Avg**.	21.9	25.4	3.5	66.5	44.9	−21.6	27.3	24.8	−2.7	49.2	50.0	0.8	11.1	2.1	−9.0	1.3	−0.8	−2.1	11.5	−4.2	−15.7	−2.8	1.0	1.8	
**SD**	10.0	10.9	11.0	8.2	3.8	8.4	11.2	10.4	11.9	15.8	9.4	15.3	16.5	10.1	13.0	10.9	11.2	3.5	11.2	6.7	11.1	9.9	5.9	9.8	

These parameters were shown in the maximum (Max.), minimum (Min.), average (Avg.), and standard deviation (SD). Pre, pre-operation; Post, post-operation; D, the difference value calculated by postoperative data minus preoperative data; ^*^, relative data compared to the healthy side were used.

### Correlation Between Various Variables and Dynamic Gait Performance

**FIGURE 3:**
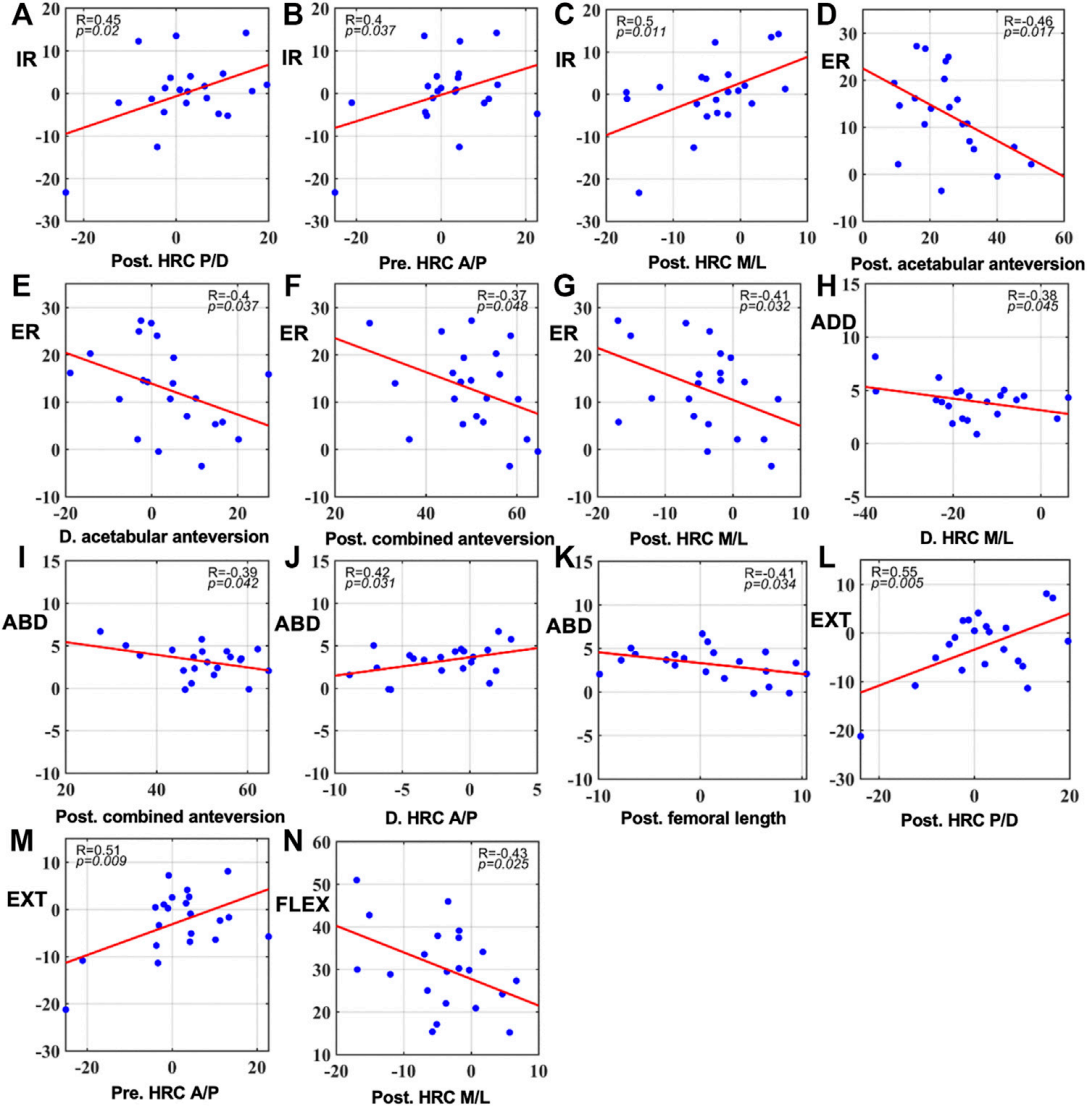
Significant correlations between gait 6-DOF ROM and preoperative and postoperative component positions as well as their changes in surgery (**(A–C)**: correlations with internal rotation. **(D–G)**: correlations with external rotation. **(H)**: correlation with adduction. **(I–K)**: correlations with abduction. **(L,M)**: correlations with extension. **(N)**: correlation with flexion. *p* < 0.05). Pre: preoperative; Post: postoperative. D: Difference calculated by postoperative data minus preoperative data. Angles were measured in degrees (**°**) and distance in millimeters (mm).

Pearson correlation coefficient analysis revealed significant correlations with gait 6-DOF ROM mainly in postoperative structures (Figure 3, *p* < 0.05). A distally, medially, and posteriorly placed hip center had high correlations with external rotation and flexion increasing (Figure 3G, Figure 3N, respectively) and internal rotation and extension decreasing (Figure 3A and Figure 3C, Figure 3L, respectively). When postoperative combined anteversion was increased, abduction was less (Figure 3I). No significant correlation was found between kinematics and demographic variables.

### Forward Stepwise Regression for Specific Contributing Factors

The forward stepwise multiple regression analysis selected a laterally placed acetabulum cup as the significant variable for internal rotation increasing (*R* ^2^ = 0.25, *p* = 0.021) (Table 3). Acetabulum anteversion increasing explained 20% of external rotation decreasing (*p* = 0.034). When taking postoperative hip center medial/lateral distance into consideration, this percent grew into 45% (*p* = 0.004, the constant changed to 21.34 and the coefficient for acetabulum anteversion changed to −0.45). A more superior hip center significantly increased extension (*R* ^2^ = 0.30, *p* = 0.010). No parameters were selected for flexion and abd-adduction. No significant demographic variables or changes in surgery were included in multiple regression models. Normality tests of the residuals in the aforementioned analysis were all passed.

**TABLE 3:** Coefficients, *R* ^2^, and *p* values of the predictors identified by forward multiple liner regression analysis.

Predictors in the model	Coefficient	R^2^	*p*
Internal rotation			
Constant	2.69		
Postoperative HRC M/L distance	0.62	0.25	0.021
External rotation			
Constant	22.51 (21.34)		
Postoperative acetabulum anteversion	−0.38 (−0.45)	0.20	0.034
+Postoperative HRC M/L distance	−0.66	0.45	0.004
Extension			
Constant	−3.40		
Postoperative HRC P/D distance	0.37	0.30	0.010

M/L, medial/lateral; P/D, proximal/distal.

## Discussion

Our study revealed significant Pearson correlations between postoperative structures and gait maximum range of motion in unilateral DDH patients after THA during gait. Further regression analysis showed that a laterally placed hip center was correlated with an increased internal rotation, which together with increased acetabular anteversion, was also associated with decreased external rotation. A higher hip center was correlated with more extension. No other parameters had significant regression models with postoperative gait ROM. These results indicated that postoperative prosthesis positions, particularly hip center positions in medial/lateral and proximal/distal directions, had a more significant impact on the gait motion range in DDH patients. In contrast, demography or preoperative status or changes in surgery had less influence.

Restoring structures to a healthy anatomic hip is the common goal for THA to reduce hip load, improve normal hip biomechanics, and support normal gait function (Galia et al., 2017). Nevertheless, such goals turned out to be a quite difficult challenge due to pathomorphology changes in some DDH patients. Compromised reconstruction goals were proposed for such patients to reduce the disturbance to lower extremity alignments and the need for adjuvant procedures such as femoral shortening osteotomy or acetabulum bone grafting, which lowered the duration of anesthesia and surgery complexity and favored postoperative recovery (Russotti and Harris, 1991; Gustke, 2004; Yu et al., 2019). Such techniques take preoperative morphology into account and conflict against common anatomic reconstruction targets. Clinical follow-up showed different results from good to poor (Traina et al., 2009; Montalti et al., 2018; Stirling et al., 2021). Karaismailoglu found asymmetry gait, especially a lower extension range in unilateral DDH patients after high hip center THA and suggested the anatomical center reconstruction technique more suitable for better walking patterns (Karaismailoglu et al., 2019a). Li reported that anatomic hip center restoration together with osteotomy allowed for safely functional limb lengthening and achieved good functional and radiographic outcomes (Li et al., 2017). Our result from both correlation and regression analysis showed that postoperative component positions had a greater impact on gait range of motion following THA, in favor of the concept that postoperative structures were crucial factors for gait motion range which should be treated with priority instead of preoperative morphology or changes in surgery. In other words, when hip structures were satisfactorily rebuilt after surgery, an ideal range of motion during gait could be assured. Further research studies were needed to quantify these hip reconstruction parameters for a more precise reconstruction guidance.

Among various postoperative structures, the rotation center position was one of the main factors influencing postoperative kinematics after THA. Nie found that cup superior displacement over 12 mm contributed to asymmetry gait patterns in patients with DDH after unilateral THA (Nie et al., 2017). Karaismailoglu suggested a lower extension range after higher hip center THA via gait analysis (Karaismailoglu et al., 2019a). This was against our findings that the higher postoperative hip center position was associated with more extension. However, our results could be possibly explained that primary osteoarthritis patients after standard THA experienced an average extension ROM of −10° during gait which was smaller than that of our results of −2.6°, while their hip centers were anatomically rebuilt in surgery (Tsai et al., 2014). In our study, we chose a slightly superior hip center in DDH patients, which was elevated by an average of 2.1 mm. If hip centers were anatomically rebuilt, extension ranges might be reduced to the level of that in standard THA. The reasons for such contradiction might lie in measurement accuracy. We adopted DFIS for motion detection and measured anatomic parameters based on 3D models, in which measurement accuracy would be elevated. Thus, our results might differ from those by gait analysis and measurement based on two-dimensional X-rays. In addition, our results also found that a laterally placed hip center counted for 25% of internal rotation increasing, while a more medial hip center resulted in external rotation increasing. It was possibly explained that a laterally placed hip center decreased femoral offset (FO), which is the moment arm of abductors (Sariali et al., 2014; Bjørdal and Bjørgul, 2015), and increased hip internal was adopted as a compensatory to restore the abductor moment arm to compensate abduction capacity as reported previously (Arnold et al., 1997). The subsequent influence on FO following changed hip center positions and its impact on gait patterns deserved further study. Moreover, it was quite clear from the literature that laterally or proximally placed hip centers increased hip loads and risks of loosening (Pagnano et al., 1996; Bicanic et al., 2009; Bonnin et al., 2011). The Harris Hip Score domains of activity of daily life and gait were also significantly reduced in these patients (Berninger et al., 2019). Interestingly, these results might be potentially related to our findings that laterally or proximally placed hip centers increased abnormal extension and internal rotation which damaged gait patterns and should be avoided. Further research studies were needed.

In our study, no correlation was found between femoral length discrepancy and gait kinematics patterns. Leg length is another consideration in DDH patients after THA. Boundary effect existed that a 10-mm discrepancy was reported to be the marginal value for gait asymmetry and a discrepancy over 20 mm deteriorated postoperative recovery, resulting in severe gait asymmetry and greater loosening risks (Kaufman et al., 1996; Bhave et al., 1999; Lai et al., 2001). Chen found that DDH patients with postoperative relative leg length discrepancy displayed significantly reduced hip range of motion, mainly extension, on the operated side compared with the healthy control group (Chen et al., 2018). In our cohort, we chose a slightly superior hip center when it was challenging to reduce or cup coverage was insufficient at the anatomic acetabulum. When reduction difficulty still existed, we preferred deeper stem fixation rather than femoral osteotomy. By such strategies, we managed to achieve nearly equal leg length after surgery with the maximum discrepancy of 10.4 mm. Since boundary effect existed, its impact on kinematics was minor, and no correlations became detectable. Vertical hip center deviations, in turn, became the dominating factor determining the range of extension. Interestingly, it was proved from the other side that within the same amount of changes less than 10 mm, vertical hip center positions had greater power in altering kinematic patterns. This indicated the importance of assuring an appropriate and precise hip center position in surgery.

The effect of acetabular orientation on maximum hip range of motion before impingement was extensively studied on computers previously (Widmer and Zurfluh, 2004; Widmer, 2020). In these studies, increasing acetabulum anteversion was correlated with decreasing external rotation, and the range of motion before impingement was limited. Although the decreasing external rotation was also found to be correlated with the increasing acetabulum anteversion in our study, it was quite different from aforementioned previous studies which focused on impingement. Impingement happened in extreme motion degrees and generally would not occur in gaits. Tsai found that internal rotation was significantly correlated with cup anteversion, while he did not give a reasonable explanation (Tsai et al., 2015). No previous studies reported whether acetabular anteversion affected the *in vivo* hip kinematics of DDH patients after THA during gait.

The results of the current study need to be interpreted in light of several limitations. First, the sample size in this study was relatively small. Our findings are preliminary which need to be confirmed by studies with a larger sample size. Second, numerous factors might influence postoperative gait kinematics patterns, such as personal gait posture. We included a wide range of potential factors, including demographic variables, preoperative and postoperative anatomy and component positions and their changes in surgery, and controlled testing conditions. The result was credible under the current setting.

## Conclusion

In conclusion, this study used DFIS to investigate the relationship of various factors, including demographic variables, preoperative, and postoperative anatomy and component positions and their changes in surgery with *in vivo* hip range of motion of the implanted side in unilateral DDH patients during gait. Significant correlations were revealed between postoperative prosthesis positions and gait range of motion. Further regression analysis showed that a laterally placed hip center was correlated with increased internal rotation and decreased external rotation. A higher hip center was correlated with more extension. It indicated that postoperative structures, particularly hip center positions, had the most significant impact on kinematics among preoperative structures and changes in surgery and should be treated with particular caution in surgery.

## Data Availability

The raw data supporting the conclusions of this article will be made available by the authors, without undue reservation, to any qualified researcher.

## Abbreviations

DDH, developmental dysplasia of the hip; THA, total hip arthroplasty; DFIS, dual fluoroscopic imaging system; CT, computed tomography; 6-DOF, six-degrees-of-freedom; APP, anterior pelvic plane; TPP, transverse pelvic plane; MSP, medial sagittal plane; ASIS, anterior–superior iliac spine; HRC, hip rotation center.
